# Agreement between commercial assays for haptoglobin and serum amyloid A in goats

**DOI:** 10.1186/s13028-017-0333-9

**Published:** 2017-10-02

**Authors:** Michał Czopowicz, Olga Szaluś-Jordanow, Marcin Mickiewicz, Agata Moroz, Lucjan Witkowski, Iwona Markowska-Daniel, Daria Reczyńska, Emilia Bagnicka, Jarosław Kaba

**Affiliations:** 1 Laboratory of Veterinary Epidemiology and Economics, Faculty of Veterinary Medicine, Warsaw University of Life Sciences, Nowoursynowska 159c, 02-776 Warsaw, Poland; 2 Division of Infectious Diseases, Department of Small Animal Diseases with Clinic, Faculty of Veterinary Medicine, Warsaw University of Life Sciences, Nowoursynowska 159c, 02-776 Warsaw, Poland; 3 Institute of Genetics and Animal Breeding, Polish Academy of Sciences, Postępu 36A, Jastrzębiec, 05-552 Magdalenka, Poland

**Keywords:** Acute phase proteins, ELISA, Small ruminants

## Abstract

Haptoglobin (Hp) and serum amyloid A (SAA) are considered as the major acute phase proteins (APPs) in goats. These APPs have been investigated in several studies during the last decade. In most studies, a colorimetric assay for Hp and a solid phase sandwich ELISA for SAA have been used for quantification. In 2015, reference intervals for APPs were determined using a new type of assay, the competitive ELISA (cELISA). Results obtained by the cELISA differed significantly from results obtained by previously used assays. The present study aimed to assess the agreement between so far used assays and cELISAs. Sera of 152 female dairy goats of two Polish national breeds were analysed. The concentration of Hp was determined using a colorimetric assay (Hp-CA) and the cELISA (Hp-cELISA), while a solid phase sandwich ELISA (SAA-sELISA) and the cELISA (SAA-cELISA) were used to measure SAA. Agreement between test results was assessed by preparing Bland–Altman plots, and analyzing 95% limits of agreement (LoA). Finally, the assays for Hp and SAA were compared using 147 and 138 serum samples, respectively, as 5 and 14 paired measurements, respectively, were excluded from agreement analyses to avoid extrapolation of Hp and SAA concentration. Measurements obtained by the Hp-CA and Hp-cELISA showed weak positive correlation (r = 0.24, P = 0.003). Limits of agreement (LoA) ranged from + 1.6 (95% CI 1.4 to 1.8) g/L to − 1.5 (95% CI − 1.7 to − 1.3) g/L. Measurements yielded by the SAA-sELISA and SAA-cELISA did not correlate (r = − 0.01, P = 0.855). LoA ranged from + 14.5 mg/L (95% CI 12.9 to 16.1) to − 8.5 mg/L (95% CI − 10.1 to − 6.9). Agreement between the two types of commercial assays for determination of Hp and SAA concentrations in goats is poor and cELISAs tend to underrate both Hp and SAA concentrations.

**Electronic supplementary material:**

The online version of this article (doi:10.1186/s13028-017-0333-9) contains supplementary material, which is available to authorized users.

## Findings

Haptoglobin (Hp) and serum amyloid A (SAA) are the major acute phase proteins (APPs) in goats [1] and a significant increase of either Hp or SAA concentrations or both has been shown in parasitic [2–4] and bacterial [5, 6] infections as well as in non-infectious conditions [7–9]. For quantification, most of these studies employed a colorimetric assay for Hp (Hp-CA) and a solid phase sandwich ELISA for SAA (SAA-sELISA). Hp levels in healthy goats used as controls in these studies consistently remained below 0.5 g/L, whereas SAA levels varied considerably from < 1 to > 150 mg/L. A common drawback to all these studies was the small sample size ranging from six to 26 individuals. In 2015, competitive ELISAs (cELISAs) were introduced for quantification of Hp (Hp-cELISA) and SAA (SAA-cELISA). By using the cELISAs on a group of 50 clinically healthy goats, Heller and Johns [10] found levels of Hp and SAA that differed from previously recognized reference values. By using the cELISAs, a Hp level of 0.4–1.2 g/L was considered normal as was a SAA level ranging from 0.4 to 2.1 mg/L. In particular, the upper limit for SAA was surprising as it was even 100-fold lower than observed before and what is regarded as normal in cattle and sheep [11]. On the other hand, in cattle and sheep Hp concentration above 1 g/L had so far been considered as the unspecific hallmark of severe inflammation [12]. The findings by Heller and Johns [10] were supported by those of another study [13], which we carried out in pregnant goats using the same cELISAs that yielded almost similar results. Therefore, we decided to assess the agreement between the so far used assays for Hp and SAA, and cELISAs.

Sera of 152 dairy female goats of Polish White Improved and Polish Fawn Improved breeds were used. The goats were kept in three herds in western Poland, where they were housed in concrete buildings on straw bedding and did not have access to pasture. Feeding was based on hay, haylage or corn silage, and wheat and oat. The goats’ age ranged from 3 to 9 years with a mean (± SD) of 5.0 ± 1.4 years. They were blood sampled in July 2014 (51 goats) and again in July 2015 (101 goats) during a routine serological monitoring for small ruminant lentivirus (SRLV) infection. Following collection, blood was kept overnight at 4 °C, then centrifuged at 3000 rpm for 10 min, and partitioned into three 2 mL serum aliquots. One sample was screened for SRLV infection using a whole-virus ELISA (ID Screen MVV/CAEV indirect—screening test; ID.vet Innovative Diagnostics, France), and the two others were frozen at − 20 °C until analysis. Blood collection was approved by the 3rd Local Ethical Committee in Warsaw (Approval No. 31/2013, 22 May 2013).

The Hp concentration was determined in each serum sample by using two commercial tests: (1) a Hp-CA (PHASE™ RANGE Haptoglobin kit, Tridelta Development Ltd., Ireland) and (2) a Hp-cELISA (Goat haptoglobin Hpt/HP ELISA kit, Cusabio Wuhan Huamei Biotech Co., Ltd., China). Sera were diluted 1:2 for the Hp-CA and 1:30,000 for the Hp-cELISA. Given that the lowest and the highest concentrations of calibrators used were 0 and 2.5 g/L, respectively in the Hp-CA, and 0 and 1 mg/L, respectively in Hp-cELISA, the lower and upper limits of standard curves ranged from 0 to 5 g/L in the Hp-CA and from 0 to 30 g/L in the Hp-ELISA. Inherent imprecision (random error, inter-assay coefficient of variation, CV%) was 5.7% for the Hp-CA and 10% for the Hp-cELISA according to manufacturers’ manual.

The SAA concentration was determined in each serum sample also by using two commercial tests: (1) a solid phase sandwich ELISA (SAA-sELISA) (PHASE™ RANGE multispecies SAA ELISA kit, Tridelta Development Ltd., Ireland;) and (2) a SAA-cELISA (Goat serum amyloid A SAA ELISA kit, Cusabio Wuhan Huamei Biotech Co., Ltd., China;). Sera were diluted 1:100 for the SAA-sELISA and undiluted sera were used in SAA-cELISA. Given that the lowest and the highest concentrations of calibrators used were 0 and 0.3 mg/L, respectively in SAA-sELISA and 0 and 8 mg/L, respectively in SAA-cELISA, the lower and upper limit of standard curves ranged from 0 to 30 mg/L in SAA-sELISA and from 0 to 8 mg/L in SAA-cELISA. CV% was 12.1% for the SAA-sELISA and 15% for the cELISA according to manufacturers’ manual.

All assays were performed and results interpreted according to manufacturers’ manuals except for sera dilution, which was chosen to fit the standard curve. The optical density was read by an Epoch Microplate Spectrophotometer (BioTek, Winooski, VT, USA) with an upper limit of 4.0. The analyses were performed in December 2016 so the samples had been stored at − 20 °C for 17 months (101 samples) or 29 months (51 samples). Before use, the assay kits had been stored in a refrigerator at 2–8 °C. All assays were performed by the same person (MC). Hp analyses were done on December 7th and SAA assays on December 8th. Serum samples were taken out of the freezer the day before testing and thawed at 4 °C in a refrigerator. Then, the required serum dilutions for the Hp assays were prepared on non-coated Nunc^®^ MicroWell™ 96 well polystyrene U-plates plates (Merck KGaA, Darmstadt, Germany), and undiluted serum samples were immediately returned to a refrigerator and stored overnight. The Hp-CA was immediately performed (took roughly 1 h), followed by the Hp-cELISA (took roughly 2.5 h). On the next day, the procedure was repeated for the SAA assays: dilution was prepared for the SAA-sELISA and undiluted sera were transferred onto non-coated polystyrene U-plates and left altogether for 1 h to reach room temperature. Then, the SAA-sELISA was performed, followed by the SAA-cELISA (each took roughly 2.5 h).

Hp and SSA concentrations yielded by each assay were reported as a median, interquartile range and range. One-sample Student’s t test and a Pearson’s linear correlation coefficient (r) were used to assess the difference between measurements yielded by assays from each pair. The inherent imprecision of both methods (CV%_both_) was calculated according to the formula: (CV%^2^_assay1_ + CV%^2^_assay2_)^1/2^, where CV%_assay1 or 2_ stood for an individual inherent imprecision (random error, inter-assay coefficient of variation) of each assay. The 95% acceptance limits based on inherent imprecision of both assays were given as: 0 ± 1.96 × CV%_both_ × the mean value of the two assays according to [14]. Agreement between test results was assessed by preparing a line of equality plot and a Bland–Altman plot (a difference against mean plot or a bias plot), and analyzing 95% limits of agreement (LoA) with their 95% confidence intervals (95% CI) according to [15]. All statistical tests were two-sided and a significance level (α) was set at 0.05. Statistical analysis was performed using Statistica 12 (StatSoft Inc., Palo Alto, CA, USA).

Seventy-five goats (49.0%) tested positive for SRLV infection and 51 of these had evident carpal arthritis (33.6% of all goats). The upper limit of reliable detection of Hp in the Hp-CA (> 5.0 g/L) was exceeded in five samples (values: 5.2, 7.0, 7.1, 10.7, and 12.1 g/L). Similar, the upper limit for SAA in the SAA-sELISA (> 30 mg/L) was exceeded in 14 samples (values: 34.0, 34.7, 35.7, 36.2, 38.0, 39.3, 41.1, 45.5 and six samples were > 47.8 mg/L). These measurements (and their counterparts in cELISAs) were therefore excluded from the analysis of assay agreement, so that these measurements in which Hp or SAA concentration was obtained by extrapolation from the standard curve were not used. Finally, the assays for Hp and SAA were compared using 147 (152 minus the aforementioned five) and 138 (152 minus the aforementioned 14) paired measurements, respectively (Table 1).

**Table 1:** Haptoglobin (Hp) and serum amyloid A (SAA) concentration in 147 and 138 goat serum samples, respectively

Acute phase protein	Unit	Concentration (median, interquartile range, range)	
		cELISA	Other assays^a^
Haptoglobin	g/L	0.47, 0.36–0.61 (0.16–1.36)	0.26, 0.24–0.31 (0.21–4.89)
Serum amyloid A	mg/L	0.46, 0.39–0.54 (0.24–2.1)	0.59, 0.41–4.10 (0.29–28.70)

^a^PHASE™ RANGE Haptoglobin kit (Tridelta Development Ltd., Ireland) for Hp, and PHASE™ RANGE multispecies SAA ELISA kit (Tridelta Development Ltd., Ireland) for SAA

The inherent imprecision of the two assays (CV%_both_) was 11.5% for Hp assays and 19.3% for SAA assays.

**Fig. 1:**
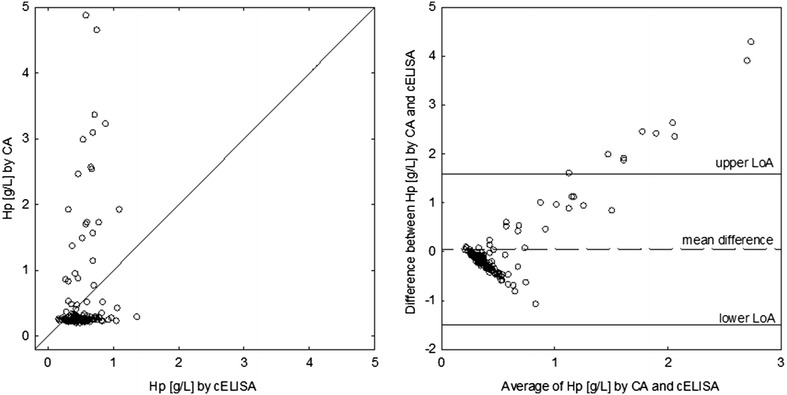
A line of equality plot and a Bland–Altman plot for 147 measurements of haptoglobin (Hp) obtained using a colorimetric assay (CA) and a competitive ELISA (cELISA) in goats. LoA stands for 95% limits of agreement

Measurements yielded by the Hp-CA and the Hp-cELISA showed weak positive correlation (r = 0.24, P = 0.003) and the mean difference did not differ significantly from 0 (0.05 g/L, 95% CI − 0.08, 0.18 g/L; P = 0.446). As a result the 95% acceptance limits based on inherent imprecision of both assays were ± 0.1 g/L. However, 95% of measurements were expected to differ between these two assays by ± 1.6 g/L, which is a much higher figure than acceptable. Along with increasing Hp concentration in a sample, the discrepancy between measurements increased, specifically the cELISA tended to underrate Hp concentration compared to the colorimetric assay (Fig. 1).

**Fig. 2:**
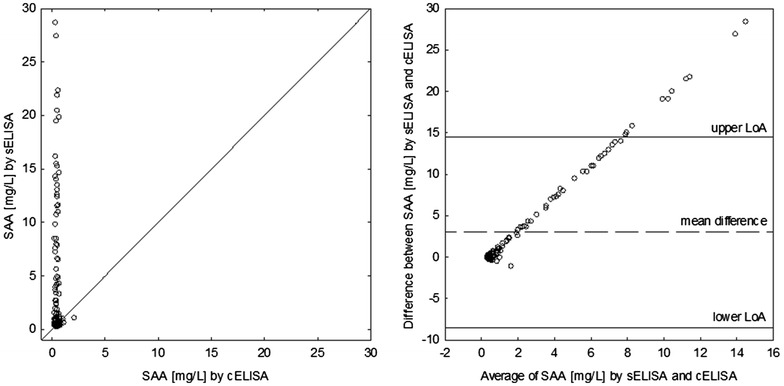
A line of equality plot and Bland–Altman plot for 138 measurements of serum amyloid A (SAA) obtained using a sandwich ELISA (sELISA) and a competitive ELISA (cELISA) in goats. LoA stands for 95% limits of agreement

The agreement between the SAA-sELISA and the SAA-cELISA was even worse. Measurements by these two assays did not correlate at all (r = − 0.01, P = 0.855) and the mean difference differed significantly from 0 (3.0 mg/L, 95% CI 2.1, 3.9 mg/L; P < 0.001). As a result, the 95% acceptance limits based on inherent imprecision of both assays were ± 0.8 mg/L. However, 95% of measurements of the SAA-sELISA were expected to be from 14.5 mg/L higher to 8.5 mg/L lower than those of the SAA-cELISA. The discrepancy between measurements was significantly higher than acceptable. It also increased with increasing SAA concentration in a sample, and again the cELISA tended to underrate the SAA concentration compared to the sELISA (Fig. 2); however, this tendency was much stronger than for the Hp assays. Furthermore, lack of correlation meant that measurements of one assay could not be predicted using measurements of the another assay. Detailed results of the four assays are presented in Additional file 1.

The disparity between results obtained by the two assays’ is unacceptable from a clinical standpoint given the reference intervals, which are much narrower than the diversity of results obtained. Obviously, the study does not determine which of the two assays that yielded true results; potentially both could be inaccurate. Therefore, further studies are needed to estimate the accuracy of both methods, preferably by comparing with other available laboratory methods.

Three issues need further discussion. First, the Hp-cELISA kit used in this study was close to expiry date (analyses done on December 8th while expired on December 14th). This might have accounted for slightly lower figures obtained by this assay; however no significant decline in the assay’s capability to quantify the substance should occur within the validity period, especially given that all three assays were stored in conditions consistent with manufacturers’ recommendations. Second, serum samples had been stored much longer than recommended (1 year for the Hp-CA and Hp-sELISA and 1 month for both cELISAs). This undoubtedly could have reduced concentrations of Hp and SAA [16, 17], however this should not affect the agreement between the assays. This study did not aim to determine the accuracy of the two assays (an extent to which they conform to the true level) but their agreement (an extent to which their results conform to each other) [18]. Even if the results on a given serum sample are utterly false, e.g. due to a time-related decrease in the substance concentration, they are supposed to remain similar. Unfortunately, they proved to be very different. Finally, the sample dilution used in the Hp-cELISA (1:30,000) was much higher than the initial dilution recommended by the manufacturer (1:500). This increased dilution was based on our previous experience [13]: the Hp-cELISA is a competitive inhibition assay in which color intensity correlates negatively with the Hp concentration, i.e. the higher Hp concentration in the sample the lighter color in the well of ELISA plate. When we used the recommended dilution of 1:500 or even dilutions several-fold higher, the wells used to remain colorless (which we interpreted as too high concentration of Hp in the well). Only at dilutions of 1:18,000 [13] or higher, wells containing different samples started to differ in color intensity. However, it must be stressed that reaching such a high dilution requires a several-step procedure and, despite the fact that we did our utmost to be precise, it may have adversely affected obtained results. One premise, which may substantiate these doubts is that in other studies [1–9], Hp level quantified using Hp-CA used to be below 0.5 g/L in healthy goats, while in the study using Hp-cELISA [10] it was between 0.4 and 1.2 g/L. The Hp-cELISA therefore appeared to overrate the Hp concentration compared to Hp-CA. Our observations were contrary. Unfortunately, the dilutions used were not reported by Heller and Johns [10]. Therefore, this issue requires further investigation.

Agreement between the two types of commercial assays determining Hp and SAA concentration in serum of goats was found to be poor and cELISAs seemed to underrate both Hp and SAA concentrations compared to the colorimetric assay and the sandwich ELISA, respectively. This needs to be taken into consideration when Hp and SAA are quantified.

## Additional file

**Additional file 1.** Detailed results obtained using four different assays for acute phase proteins in goats. SRLV—Small ruminant lentivirus; CAE—caprine arthritis-encephalitis; Hp-CA—colorimetric assay for haptoglobin (Hp); SAA-sELISA—sandwich ELISA for serum amyloid A (SAA); Hp-cELISA—competitive ELISAs for Hp; SAA-cELISA—competitive ELISA for SAA.

## Abbreviations

APP: acute phase protein
CAE: caprine arthritis-encephalitis
CI: confidence interval
CV%_both_: inherent imprecision of both assays
CV%_assay1/2_: inherent imprecision of one assay
Hp: haptoglobin
Hp-CA: a colorimetric assay for haptoglobin
Hp-cELISA: competitive ELISA for haptoglobin
LoA: limits of agreement
SAA: serum amyloid A
SAA-cELISA: competetive ELISA for serum amyloid A
SAA-sELISA: sandwich ELISA for serum amyloid A
SRLV: small ruminant lentivirus
