# Supplementary material for: Agreement between commercial assays for haptoglobin and serum amyloid A in goats
Source: Acta Vet Scand. 2017 Oct 2;59:65. doi: 10.1186/s13028-017-0333-9 (PMC5625724; doi:10.1186/s13028-017-0333-9)
Supplement: Supplementary file 1 — Additional file 1. Detailed results obtained using four different assays for acute phase proteins in goats. SRLV—Small ruminant lentivirus; CAE—caprine arthritis-encephalitis; Hp-CA—colorimetric assay for haptoglobin (Hp); SAA-sELISA—sandwich ELISA for serum amyloid A (SAA); Hp-cELISA—competitive ELISAs for Hp; SAA-cELISA—competitive ELISA for SAA. [file 13028_2017_333_MOESM1_ESM.docx]

**Additional file 1. Detailed results obtained using four different assays for acute phase proteins in goats**

SRLV – Small ruminant lentivirus; CAE – caprine arthritis-encephalitis; Hp-CA – colorimetric assay for haptoglobin (Hp); SAA-sELISA – sandwich ELISA for serum amyloid A (SAA); Hp-cELISA – competitive ELISAs for Hp; SAA-cELISA – competitive ELISA for SAA

| Goat no. | Herd no. | SRLV serological status | Clinical signs of CAE | Months of storage at -20 °C | Hp-CA [g/L] | Hp-cELISA [g/L] | SAA-sELISA [mg/L] | SAA-cELISA [mg/L] |
| --- | --- | --- | --- | --- | --- | --- | --- | --- |
| 1 | 1 | Positive | Present | 29 | 3.24 | 0.88 | 3.67 | 0.35 |
| 2 |  |  |  |  | 0.43 | 1.06 | 8.44 | 0.25 |
| 3 |  |  |  |  | 0.29 | 0.35 | 1.50 | 0.29 |
| 4 |  |  |  |  | 0.30 | 1.36 | 14.26 | 0.37 |
| 5 |  |  |  |  | 0.42 | 0.43 | 19.80 | 0.70 |
| 6 |  |  |  |  | 2.57 | 0.65 | 0.65 | 0.43 |
| 7 |  |  |  |  | 5.18* | 0.54 | 21.93 | 0.49 |
| 8 |  |  |  |  | 2.47 | 0.47 | 22.31 | 0.57 |
| 9 |  |  |  |  | 0.49 | 0.36 | 11.62 | 0.63 |
| 10 |  |  |  |  | 1.57 | 0.68 | 47.76* | 0.52 |
| 11 |  |  |  |  | 0.34 | 0.41 | 4.29 | 0.65 |
| 12 |  |  |  |  | 0.52 | 0.83 | 3.96 | 0.36 |
| 13 |  |  |  |  | 0.54 | 0.31 | 28.70 | 0.31 |
| 14 |  |  |  |  | 10.67* | 0.49 | 47.76* | 0.43 |
| 15 |  |  |  |  | 0.83 | 0.31 | 12.61 | 0.48 |
| 16 |  |  |  |  | 0.26 | 0.45 | 7.99 | 0.43 |
| 17 |  |  |  |  | 0.89 | 0.46 | 20.43 | 0.48 |
| 18 |  |  |  |  | 3.10 | 0.68 | 1.13 | 0.55 |
| 19 |  |  |  |  | 0.29 | 0.45 | 13.03 | 0.54 |
| 20 |  |  |  |  | 7.05* | 0.79 | 16.15 | 0.37 |
| 21 |  |  |  |  | 0.35 | 0.36 | 41.09* | 0.41 |
| 22 |  |  |  |  | 0.24 | 0.29 | 0.51 | 0.83 |
| 23 |  |  |  |  | 1.38 | 0.37 | 13.49 | 0.49 |
| 24 |  |  |  |  | 0.30 | 0.64 | 1.54 | 0.48 |
| 25 |  |  |  |  | 0.35 | 0.82 | 0.75 | 0.63 |
| 26 |  |  |  |  | 0.31 | 0.54 | 4.75 | 0.39 |
| 27 |  |  |  |  | 0.27 | 0.45 | 0.97 | 1.07 |
| 28 |  |  |  |  | 0.31 | 0.41 | 7.26 | 0.32 |
| 29 |  |  |  |  | 0.31 | 0.33 | 1.00 | 0.32 |
| 30 |  |  |  |  | 1.73 | 0.77 | 36.16* | 0.53 |
| 31 |  |  |  |  | 1.49 | 0.53 | 39.26* | 0.71 |
| 32 |  |  |  |  | 0.25 | 0.46 | 15.30 | 0.47 |
| 33 |  |  |  |  | 0.30 | 0.77 | 14.68 | 0.67 |
| 34 |  |  |  |  | 0.27 | 0.46 | 0.60 | 1.12 |
| 35 |  |  |  |  | 3.37 | 0.72 | 4.84 | 0.58 |
| 36 |  |  |  |  | 0.27 | 0.72 | 2.39 | 0.43 |
| 37 |  |  |  |  | 0.87 | 0.27 | 19.44 | 0.39 |
| 38 |  |  |  |  | 0.26 | 0.33 | 0.53 | 0.60 |
| 39 |  |  |  |  | 0.28 | 0.44 | 2.71 | 0.35 |
| 40 |  |  |  |  | 0.25 | 0.57 | 15.46 | 0.43 |
| 41 |  |  |  |  | 1.71 | 0.59 | 3.40 | 0.47 |
| 42 |  |  |  |  | 0.30 | 0.36 | 9.84 | 0.38 |
| 43 |  |  |  |  | 0.29 | 0.72 | 3.29 | 0.69 |
| 44 |  |  |  |  | 4.65 | 0.74 | 0.65 | 0.28 |
| 45 |  |  |  |  | 3.00 | 0.54 | 34.72* | 0.34 |
| 46 |  |  |  |  | 0.31 | 0.43 | 38.04* | 0.35 |
| 47 |  |  |  |  | 7.12* | 0.49 | 47.76* | 0.37 |
| 48 |  |  |  |  | 0.33 | 0.41 | 8.51 | 0.46 |
| 49 |  |  |  |  | 0.28 | 0.79 | 0.45 | 0.48 |
| 50 |  |  |  |  | 0.25 | 0.71 | 0.39 | 0.49 |
| 51 |  |  |  |  | 0.30 | 0.73 | 0.40 | 0.48 |
| 52 | 2 | Negative | Absent | 17 | 0.24 | 0.83 | 0.35 | 0.40 |
| 53 |  |  |  |  | 0.30 | 0.44 | 0.44 | 0.33 |
| 54 |  |  |  |  | 12.10* | 0.42 | 47.76* | 0.41 |
| 55 |  |  |  |  | 0.53 | 0.60 | 47.76* | 0.90 |
| 56 |  |  |  |  | 0.24 | 0.47 | 0.85 | 0.36 |
| 57 |  |  |  |  | 0.26 | 0.57 | 1.18 | 0.66 |
| 58 |  |  |  |  | 0.26 | 0.46 | 0.39 | 0.56 |
| 59 |  |  |  |  | 0.25 | 0.54 | 0.50 | 0.74 |
| 60 |  |  |  |  | 0.25 | 0.92 | 0.43 | 0.59 |
| 61 |  |  |  |  | 0.25 | 0.36 | 1.98 | 0.34 |
| 62 |  |  |  |  | 0.24 | 0.60 | 0.44 | 0.46 |
| 63 |  |  |  |  | 0.26 | 0.55 | 0.38 | 0.44 |
| 64 |  |  |  |  | 0.25 | 0.37 | 0.41 | 0.44 |
| 65 |  |  |  |  | 0.25 | 0.67 | 0.35 | 0.55 |
| 66 |  |  |  |  | 0.25 | 0.61 | 0.83 | 0.41 |
| 67 |  |  |  |  | 1.15 | 0.68 | 0.43 | 0.63 |
| 68 |  |  |  |  | 0.28 | 0.74 | 0.43 | 0.41 |
| 69 |  |  |  |  | 0.27 | 0.17 | 0.57 | 0.33 |
| 70 |  |  |  |  | 0.23 | 0.47 | 0.37 | 0.38 |
| 71 |  |  |  |  | 0.26 | 0.48 | 0.35 | 0.51 |
| 72 |  |  |  |  | 0.34 | 0.55 | 12.41 | 0.55 |
| 73 |  |  |  |  | 0.24 | 0.59 | 0.70 | 0.68 |
| 74 |  |  |  |  | 0.22 | 0.40 | 0.48 | 0.50 |
| 75 |  |  |  |  | 0.25 | 0.71 | 5.63 | 0.47 |
| 76 |  |  |  |  | 0.29 | 0.98 | 1.01 | 0.75 |
| 77 |  |  |  |  | 0.29 | 0.41 | 1.01 | 0.26 |
| 78 |  |  |  |  | 0.27 | 0.34 | 0.78 | 0.39 |
| 79 |  |  |  |  | 0.27 | 0.27 | 0.40 | 0.35 |
| 80 |  |  |  |  | 2.55 | 0.67 | 6.61 | 0.50 |
| 81 |  |  |  |  | 0.29 | 0.50 | 0.53 | 0.33 |
| 82 |  |  |  |  | 1.92 | 1.08 | 45.54* | 0.54 |
| 83 |  |  |  |  | 0.25 | 0.64 | 0.54 | 0.40 |
| 84 |  |  |  |  | 0.78 | 0.69 | 47.76* | 0.29 |
| 85 |  |  |  |  | 0.27 | 0.63 | 0.39 | 0.26 |
| 86 |  |  |  |  | 0.26 | 0.61 | 0.82 | 0.32 |
| 87 |  |  |  |  | 0.26 | 0.64 | 0.57 | 0.35 |
| 88 |  |  |  |  | 0.25 | 0.52 | 0.51 | 0.24 |
| 89 |  |  |  |  | 0.27 | 0.23 | 0.49 | 0.30 |
| 90 |  |  |  |  | 1.93 | 0.32 | 27.40 | 0.45 |
| 91 |  |  |  |  | 1.73 | 0.60 | 10.97 | 0.63 |
| 92 |  |  |  |  | 4.89 | 0.58 | 6.51 | 0.62 |
| 93 |  |  |  |  | 0.96 | 0.42 | 35.70* | 0.47 |
| 94 |  |  |  |  | 0.21 | 0.49 | 10.74 | 0.45 |
| 95 |  |  |  |  | 0.24 | 0.53 | 1.42 | 0.42 |
| 96 |  |  |  |  | 0.26 | 0.59 | 0.45 | 0.32 |
| 97 |  |  |  |  | 0.26 | 0.26 | 0.35 | 0.43 |
| 98 |  |  |  |  | 0.21 | 0.43 | 0.31 | 0.46 |
| 99 |  |  |  |  | 0.23 | 0.43 | 0.45 | 0.44 |
| 100 |  |  |  |  | 0.25 | 0.43 | 0.91 | 0.46 |
| 101 |  |  |  |  | 0.25 | 0.50 | 0.39 | 0.48 |
| 102 |  |  |  |  | 0.26 | 0.49 | 2.32 | 0.46 |
| 103 | 3 | Positive | Absent | 17 | 0.24 | 0.49 | 0.58 | 0.50 |
| 104 |  |  |  |  | 0.25 | 0.76 | 1.41 | 0.73 |
| 105 |  |  |  |  | 0.26 | 0.56 | 0.34 | 0.40 |
| 106 |  |  |  |  | 0.26 | 0.52 | 0.31 | 0.39 |
| 107 |  |  |  |  | 0.24 | 0.44 | 0.46 | 0.53 |
| 108 |  |  |  |  | 0.23 | 0.55 | 0.44 | 0.57 |
| 109 |  |  |  |  | 0.22 | 0.64 | 0.73 | 0.55 |
| 110 |  |  |  |  | 0.23 | 0.56 | 0.34 | 0.44 |
| 111 |  |  |  |  | 0.25 | 0.37 | 0.49 | 0.40 |
| 112 |  |  |  |  | 0.24 | 0.30 | 0.38 | 0.44 |
| 113 |  |  |  |  | 0.24 | 0.33 | 4.10 | 0.46 |
| 114 |  |  |  |  | 0.24 | 0.48 | 0.38 | 0.66 |
| 115 |  |  |  |  | 0.24 | 0.35 | 4.91 | 0.54 |
| 116 |  |  |  |  | 0.24 | 0.51 | 0.38 | 0.46 |
| 117 |  |  |  |  | 0.24 | 0.34 | 4.23 | 0.52 |
| 118 |  |  |  |  | 0.48 | 0.44 | 0.47 | 0.60 |
| 119 |  |  |  |  | 0.24 | 0.55 | 0.36 | 0.57 |
| 120 |  |  |  |  | 0.24 | 0.27 | 0.42 | 0.41 |
| 121 |  |  |  |  | 0.24 | 0.47 | 0.36 | 0.47 |
| 122 |  |  |  |  | 0.30 | 0.43 | 0.34 | 0.53 |
| 123 |  |  |  |  | 0.37 | 0.28 | 0.46 | 0.52 |
| 124 |  |  |  |  | 0.24 | 0.30 | 0.70 | 0.56 |
| 125 |  |  |  |  | 0.23 | 0.36 | 0.38 | 0.50 |
| 126 |  |  |  |  | 0.24 | 0.40 | 0.36 | 0.50 |
| 127 |  | Negative | Absent | 17 | 0.25 | 0.52 | 0.39 | 0.37 |
| 128 |  |  |  |  | 0.27 | 0.57 | 0.46 | 0.27 |
| 129 |  |  |  |  | 0.23 | 0.19 | 1.00 | 0.32 |
| 130 |  |  |  |  | 0.24 | 0.36 | 0.51 | 0.45 |
| 131 |  |  |  |  | 0.25 | 0.36 | 0.35 | 0.38 |
| 132 |  |  |  |  | 0.25 | 0.55 | 0.63 | 0.30 |
| 133 |  |  |  |  | 0.25 | 0.40 | 0.51 | 0.45 |
| 134 |  |  |  |  | 0.26 | 0.34 | 0.45 | 0.47 |
| 135 |  |  |  |  | 0.25 | 0.46 | 0.38 | 0.46 |
| 136 |  |  |  |  | 0.23 | 0.82 | 0.45 | 0.54 |
| 137 |  |  |  |  | 0.23 | 0.47 | 0.41 | 0.50 |
| 138 |  |  |  |  | 0.24 | 1.05 | 0.36 | 0.75 |
| 139 |  |  |  |  | 0.27 | 0.28 | 0.54 | 0.34 |
| 140 |  |  |  |  | 0.22 | 0.31 | 0.41 | 0.53 |
| 141 |  |  |  |  | 0.23 | 0.42 | 0.37 | 0.42 |
| 142 |  |  |  |  | 0.24 | 0.34 | 0.36 | 0.59 |
| 143 |  |  |  |  | 0.24 | 0.58 | 0.52 | 0.48 |
| 144 |  |  |  |  | 0.22 | 0.44 | 0.40 | 0.64 |
| 145 |  |  |  |  | 0.24 | 0.29 | 7.82 | 0.48 |
| 146 |  |  |  |  | 0.24 | 0.40 | 33.96* | 1.08 |
| 147 |  |  |  |  | 0.24 | 0.39 | 1.08 | 2.14 |
| 148 |  |  |  |  | 0.31 | 0.59 | 11.51 | 0.53 |
| 149 |  |  |  |  | 0.25 | 0.18 | 0.34 | 0.39 |
| 150 |  |  |  |  | 0.24 | 0.23 | 0.31 | 0.37 |
| 151 |  |  |  |  | 0.24 | 0.32 | 1.47 | 0.46 |
| 152 |  |  |  |  | 0.24 | 0.33 | 0.29 | 0.43 |

* Measurements exceeding the upper limit of reliable detection (obtained by extrapolation from the standard curve) and hence dropped from the analysis
